# Vector magnetocardiography using compact optically-pumped magnetometers

**DOI:** 10.1016/j.heliyon.2024.e29092

**Published:** 2024-04-01

**Authors:** Shengran Su, Zhenyuan Xu, Xiang He, Guoyi Zhang, Haijun Wu, Yalan Gao, Yueliang Ma, Chanling Yin, Yi Ruan, Kan Li, Qiang Lin

**Affiliations:** a Zhejiang Provincial Key Laboratory and Collaborative Innovation Center for Quantum Precision Measurement, College of Science, Zhejiang University of Technology, Hangzhou, 310000, China; b Hangzhou Q-MAG Technology Co.,Ltd., Hangzhou, 310000, China

**Keywords:** Vector magnetocardiography, SERF magnetometer, Multichannel measurement

## Abstract

Optically pumped magnetometers can provide functionality for bio-magnetic field detection and mapping. This has attracted widespread attention from researchers in the biomedical science field. Magnetocardiography has been proven to be an effective method for examining heart disease. Notably, vector magnetocardiography obtains more spatial information than the conventional method by only taking a component that is perpendicular to the chest surface. In this work, a spin-exchange-relaxation-free (SERF) magnetometer with a compact size of 14 mm × 25 mm × 90 mm was developed. The device has a high sensitivity of 25 fT/ $\sqrt{\text{Hz}}$. Meanwhile, in the multichannel working mode, synchronous sensor manipulation and data acquisition can be achieved through our control software without additional data acquisition boards. Since a typical SERF magnetometer only responds to dual-axis magnetic fields, two sensors are orthogonally arranged to form a vector detection channel. Our system consists of seven channels and allows 7 × 9 vector MCG mapping by scanning. High-quality heart vector signals are measured, and P peak, QRS peak, and T peak can be distinguished clearly. To better demonstrate the vectorial information, a vector scatter plot form is also provided. Through a basic bio-electric current model, it demonstrates that triaxial MCG measurements capture a richer spatial current information than traditional uniaxial MCG, offering substantial diagnostic potential for heart diseases and shedding more light on the inversion of cardiac issues.

## 1 Introduction

Optically pumped magnetometers (OPMs) have demonstrated the potential to applied to bio-magnetic field detection [1] for tasks such as magnetocardiography (MCG) [[2], [3], [4]], magnetoencephalography (MEG) [5,6], and magnetic-nanoparticle modified drug tracking [7]. With the rapid development of OPMs, a sensitivity level of 10 fT/ $\sqrt{\text{Hz}}$ and a sensor size in the centimeter scale have been achieved with commercially available devices [8]. Their high sensitivity and compact size make it possible to build OPM-based bio-magnetic field detection instruments, replacing the conventional ones with superconducting quantum interference devices (SQUIDs). In previous decades, commercial MCG instruments with OPMs were developed [2,9].

In comparison with the SQUIDs MCG instrument, OPMs have a similar level of sensitivity without the requirement for cryogenically cooled operating conditions (usually, liquid-helium-cooled). OPM-MCG instruments have the advantages of a smaller volume and lower instrument maintenance cost [10,11]. Meanwhile, improved spatio-temporal resolution of OPM-MCG is beneficial to discover abnormalities in the electrical physiological events of the heart. The spatial resolution is not only the physical spatial resolution of the sensor, but also the discrimination ability of magnetic field sources. As the magnetic field amplitude decays according to a power law with the distance from a field source, improved signal detection can be achieved when sensors are moved closer to the target. Generally, OPM has a closer detection distance compared to SQUID [11,12]. On the other hand, the temporal resolution is defined as the minimum time interval between two electrophysiological events to be detected as distinct from each other. And it is different from a sensor-level definition that would for example be based on the bandwidth of the device. Typically, the cardiac magnetic signals have different characteristic peaks (PQRST), which are derived from the electrophysiological events at different times within a heartbeat cycle. Therefore, by analyzing the waveform of cardiac magnetic signals, we can understand the excitation state of myocardial cells and the functional status of the heart. With higher temporal resolution, it is possible to accurately obtain the time interval between various electrophysiological events, thereby more accurately understanding the functional status of the heart [12].

In contrast to traditional electrocardiogram techniques, the diagnostic approach using MCG signals presents a notable advantage by minimizing distortions caused by the intricate conductivity distribution within the tissues between the heart's anatomical position and the skin surface. This characteristic can enhance the precision of cardiac source localization and facilitates the establishment of comprehensive cardiac models [13,14]. The utilization of MCG as a diagnostic technique holds promising advantages for the early detection of myocardial ischemia and other indicative symptoms of heart disease [15]. In most instances, MCG measurements solely record the magnetic field component that is perpendicular to the chest surface [3]. At the typical stand-off distances of several centimeters for SQUID-MCG, the orthogonal components tend to be weak, so that the radial field component approximates the total field. With closer detection distance, OPMs are able to measure multiple field components to extract additional spatial information. Furthermore, if the magnetic field vector simultaneously changes in both direction and magnitude, the magnetic field peak may shift in uniaxial measurement, which may cause difficulties in accurately interpreting the electrical physiological activities of the heart. Therefore, the vectorial measurement in the magnetic field emanating from the heart can enhance the quality of bio-magnetic signals [5,6]. It confers a significant advantage in precisely determining the localization of lesions and solving the forward and the inverse problems in MCG [5,14], such as tracing the cardiac current flow inside the thorax [14]. In clinical research, vector MCG has important value in the diagnosis and treatment of myocardial inflammation. For example, in patients with myocarditis receiving immunosuppressive treatment, vector SQUID-MCG can detect changes in myocardial inflammation as early as the seventh day of treatment, while echocardiography requires 30 days of treatment to observe corresponding improvement. Therefore, it can provide clinicians with earlier and more accurate diagnostic evidence, which is helpful for early intervention and treatment of myocardial inflammation [15]. In the application of fetal MCG (fMCG), to avoid the signal influence affected by fetal orientation and movement, vector MCG is potentially useful for standardization of the fMCG waveforms and to provide a more complete and accurate analysis of fetal arrhythmias [16].

Multiple approaches have been proposed to obtain the vectorial cardiac signals. In previous reports, most of the vector measurements of MCG were achieved using SQUID [15,17]. In addition to SQUID, there are other technologies that have also attempted to achieve vector measurement. Wang et al. pioneered the utilization of a tunnel magnetoresistance sensor with 25.7 pT/√Hz to measure the triaxial components of the MCG signals [18]. Sengottuvel, S et al. provided a scheme for capturing MCG signals in three orthogonal directions of the human chest using a fluxgate magnetometer [19]. Their approach involved employing electrocardiographic signals and another fluxgate magnetometer as a reference to process the magnetocardiac signals.

We demonstrated the achievement of a vector MCG with a compact elliptically polarized laser-pumped Mx atomic magnetometer. The sensor sensitivity was around 300 fT/ $\sqrt{\text{Hz}}$ after optimization, which meets the requirement for an MCG [2]. When pursuing a higher level of sensitivity, the most common type of OPM is the so-called spin-exchange-relaxation-free (SERF) atomic magnetometers. These magnetometers have a state-of-the-art sensitivity level of 0.16 fT/ $\sqrt{\text{Hz}}$ [20]. However, a typical SERF magnetometer only responds to dual-axis magnetic fields and cannot respond to the component parallel to the pump light direction. Osborne et al. carried out a two-beam project to form a compact triaxial sensor [21], and demonstrated a four-channel vector OPM-MCG system. In this type triaxial sensor, the laser beam passes through a beam splitter and the two resultant beams enter the same cell in orthogonal directions without overlap. In other words, there are two orthogonal individual SERF magnetometers that share the same vapor cell. Herein, on the basis of dual-axis SERF magnetometers, we provide a novel strategy to build a vector MCG system. The use of a dual-axis SERF atomic magnetometer can circumvent several challenges in construction of a three-axis magnetometer, such as reducing the size and and complexity of the device, mitigating the susceptibility to crosstalk in three-axis configurations, and overcoming the difficulty in improving sensitivity [[22], [23], [24]]. In this work, compact SERF magnetometers are developed with a sensitivity level of 25 fT/ $\sqrt{\text{Hz}}$ (the mean noise floor over 60–70Hz) and a size of 14 mm × 25 mm × 90 mm. The distance between the detection surface and the vapor cell center can be as low as 6 mm to obtain a higher signal-to-noise ratio. The sensor's physical spatial resolution is 3 mm × 3 mm × 3 mm and is dependent on the vapor cell size. Control software is also developed to manipulate multiple sensors simultaneously and achieve synchronous data acquisition. Our MCG system consists of 7 channels, and each channel has two sensor units arranged orthogonally. One detects the normal direction component, $B_{z}$, as well as one tangential direction component, $B_{x}$. The other detects the other tangential direction component, $B_{y}$.

## 2 Materials and methods

**Fig. 1:**
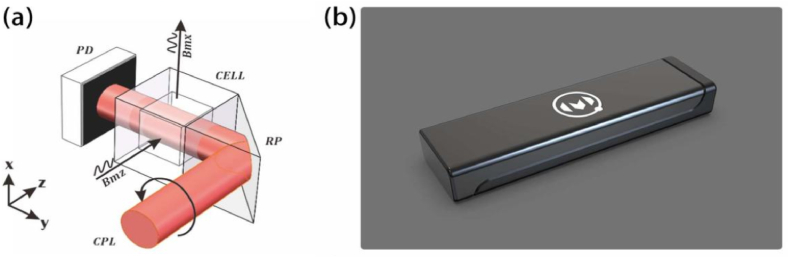
**(a)** The setup of the SERF magnetometer used in our work. RP: reflecting prism. CPL.

Our sensor works in dual-axis mode, based on the single-beam absorption SERF principle, as shown in Fig. 1 (a). A glass vapor cell (3 mm × 3 mm × 3 mm) is used to contain the ^87^Rb vapor and nitrogen buffer gas. A miniature low-noise heater is directly attached to the cell wall and heats the cell to obtain a high atomic number density. A single-mode vertical-external-cavity surface-emitting laser (VCSEL) is tuned to a D1 transition of ^87^Rb, which is around 795 nm. A circularly polarized light (CPL) beam is generated through a small optics element set. Around the vapor cell, there are three orthogonal pairs of magnetic coils that compensate for the magnetic field to generate a zero field inside the cell. Meanwhile, the set of coils in the x and z directions produces modulation fields that cooperate with the lock-in amplifier. Based on the aforementioned design approach, the sensor head features a compact structural design, resulting in a final assembled appearance as shown in Fig. 1(b), with external dimensions of 14 mm × 25 mm × 90 mm.

circularly polarized light. $B_{mx},B_{mz}$: modulation field in x and z direction. **(b)** Image of the sensor head.

Eq. (1) describes the interaction between atoms and photons in a single-beam absorption magnetometer. The average photon absorption rate per atom, ⟨Γ⟩, is related to the photon polarization of the pump light, s; the average electron angular momentum, ⟨S⟩; and the photon absorption rate, $R_{abs}$. Here, $R_{abs}$ depends on the photon frequency, υ, and it is the summation of the product of the photon absorption cross-section, σ(υ), and the total flux of photons, Φ(υ).

(1) $$\left\langle \Gamma \right\rangle =\left(1-2s∙\left\langle S\right\rangle \right)R_{abs}\left(\upsilon \right)=\left(1-2s∙\left\langle S\right\rangle \right)\int \sigma \left(\upsilon \right)\Phi \left(\upsilon \right)$$

In our system, the pump light is in the y direction and is circularly polarized, which means

s = 1. The polarization of the alkali metal atoms, P, can be defined as 2 ⟨S⟩, which implies that the change of absorption reflects the change in the polarization state. As shown in Eq. (2), P is related to the external magnetic field, **B**. Therefore, **B** can be measured through photon absorption detection. When the atoms experience the external magnetic field, the change in P will follow Eq. (2)

(2) $$\frac{d}{dt}P=\frac{1}{q}\left[\gamma B\times P+R_{op}\left(s-P\right)-R_{rel}P\right]$$

where γ is the gyromagnetic ratio of the electron, q is the nuclear slowing-down factor, $R_{\text{op}}$ is the pumping rate of the pump beam, and the $R_{\text{rel}}$ is the relaxation rate of the atoms. Since the magnetometer works in the SERF condition, the external static magnetic field is close to zero. Meanwhile, the modulation field is applied in the x and z directions, and the modulation field amplitude is $B_{m}$, and the frequency is $\omega_{m}$. $B_{mx}$ = $B_{m}$ sin($\omega_{m}$ t) and $B_{mz}$ = $B_{m}$ cos ($\omega_{m}$ t). It can be derived that the polarization in the y direction, $P_{y}$, can be approximated as shown in Eq. (3)

(3) $$P_{y}\propto P_{0}J_{0}\left(\Omega_{m}\right)J_{1}\left(\Omega_{m}\right)\left[\cos\left(\omega_{m}t\right)B_{x}+\sin\left(\omega_{m}t\right)B_{z}\right]$$

where $P_{0}=R_{op}/\left(R_{op}+R_{rel}\right)$, $J_{0}$ and $J_{1}$ are the zero and first-order Bessel functions, respectively. The variable $\Omega_{m}$ equals ${\gamma B}_{m}/{q\omega }_{m}$, and $B_{x}$ and $B_{z}$ are the measured fields. Through lock-in amplifier data processing, $B_{x}$ and $B_{z}$ can be demodulated.

**Fig. 2:**
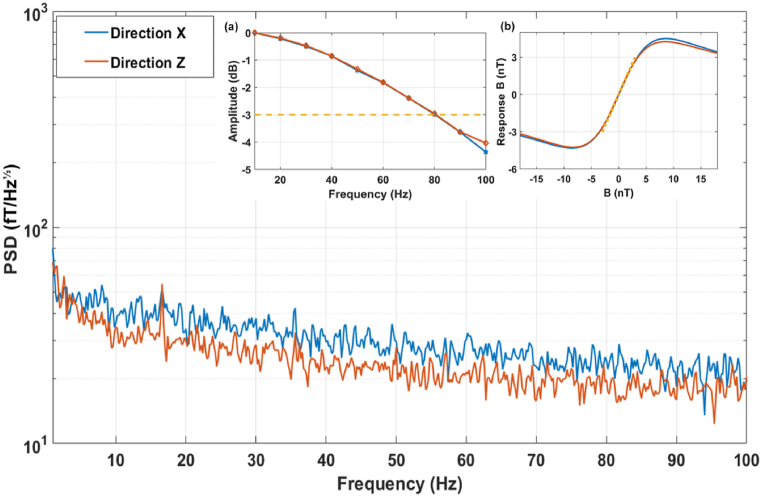
The power spectral density of our sensor covered the range of 1–100 Hz. The noise floor was around 25 fT/ $\sqrt{\text{Hz}}$. Inset (a) shows the frequency response, where the bandwidth of -3dB was about 80 Hz. Inset (b) shows the responses of the x and z axes with respect to the magnetic field in the x and z directions, where the dynamic range was around ± 3 nT.

In order to measure the sensitivity of OPMs, a sensor is located in a magnetically shielded barrel (MSB) with an internal diameter of 1.2 m. This MSB allows a background field of approximately 20 nT. In this study, the OPM signal was recorded at a sample frequency of 1 kHz for 30 s. The data were separated into 5 s segments. The power spectral density (PSD) of each data segment was calculated over the range of 0–100 Hz. The average PSD values for these segments are plotted in Fig. 2, where the OPM noise floor is around 25 fT/ $\sqrt{\text{Hz}}$ (the mean noise floor over 60–70Hz). The frequency response is plotted in Fig. 2 inset (a), where the bandwidth of −3 dB was about 80 Hz. The responses of the x and z axes with respect to the magnetic field in the x and z directions are shown in the inset (b) of Fig. 2, and a dynamic range of around ± 3 nT was obtained.

**Fig. 3:**
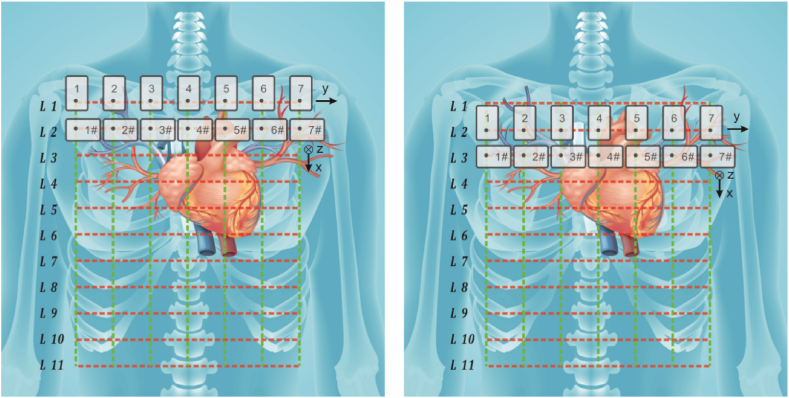
Two groups of sensors were arranged in two adjacent lines. In group 1, the y direction signals were measured, and in Group 2, the z and x direction signals were measured. After one scanning step, the sensor array was moved to the next grid line. Throughout the scanning processing, vector MCG signals from L2 to L10 were recorded to produce a 7 × 9 data matrix.

In order to scan the vector MCG, 14 OPMs were arranged as shown in (see Fig. 3). There were 2 groups of OPMs: Group 1, labeled No.1–7, was used to record the signals in the y direction; Group 2, labeled No.1#∼7 #, was used to record the signals in the z and x directions. Above each group, there were two OPMs situated 10 cm away in the z direction that were used to conduct the background vectorial magnetic field detection. The measuring region was meshed as shown in Fig. 3 to generate 11 lines in the x direction and 7 lines in the y direction. The space between each line in the x or y direction was 4 cm. The detection point of each sensor was located in the grid point. In the initial state, Group 1 was located in L1, and Group 2 was located in L2. At scanning step 1, the MCG signals in the z and x direction were recorded for group 2 in L2. At the next step, the human body was moved 4 cm in the x direction, and Group 1 was located in L2, and Group 2 was located in L3. In this step, the MCG signals in the y direction were recorded for group 1 at L2, and the z and x direction signals were recorded for group 2 on the next line, L3. Through these 2 steps, MCG signal detection was completed in three directions. We repeated the above steps until Group 1 was located in L10. Finally, the vector MCG signals from L2 to L10 were recorded; therefore, a data matrix, 7 × 9 in size, was obtained.

**Fig. 4:**
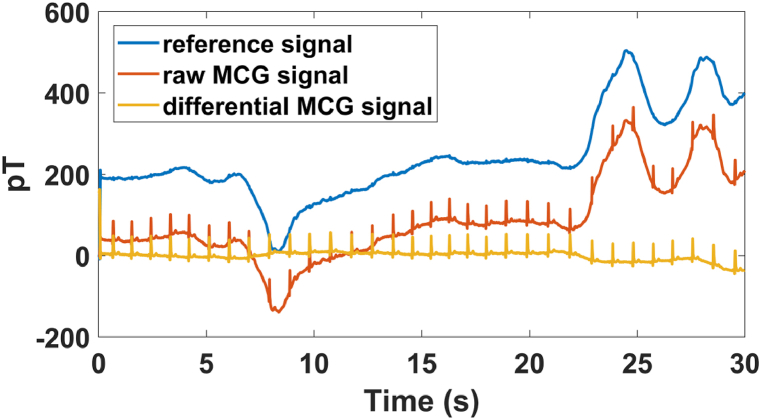
Comparison of the raw MCG signal and the differential signal.

During the measurements, one side of the MSB was open, so the external magnetic field disturbance resulted in a low-frequency shift in the signal. Fig. 4 shows the results of the detected z direction signal. The magnetic field shift in this direction was greater than 300 pT inside the MSB. Here, a common approach, the differential detection method, was carried out to diminish the common-mode interference in this detection system. It was found that the signal fluctuation was greatly reduced, and the data shift was effectively suppressed, as shown by the results presented in Fig. 4.

**Fig. 5:**
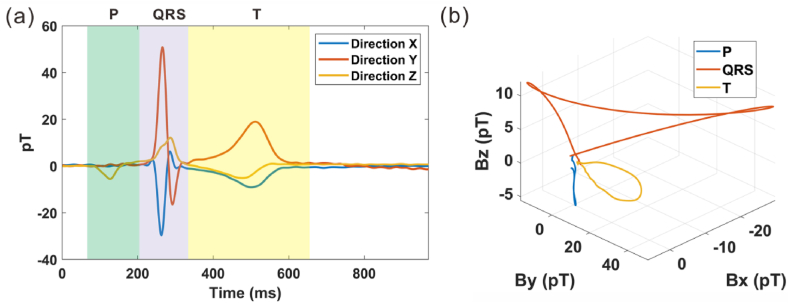
(a) Illustrates the time-domain MCG signals along the x, y, and z axes captured within a complete heartbeat cycle, while (b) presents a vector MCG scatter plot segmented into three distinct parts: the P peak in blue, the QRS peak in red, and the T peak in orange.

A single cycle of the heart signal with time for a single detection point is demonstrated in Fig. 5 (a). Due to the high sensitivity of our OPMs, the details of the signals were well preserved at a high resolution, and the P peak, QRS peak, and T peak could be distinguished effectively. However, it is hard to reflect the vector direction information in this plot form. In our previous work, we gave a different demonstration of vector MCG signals, the scatter plot, where the signal data were put into the vector space [2]. In the scatter plot shown in Fig. 5 (b), the vector rotation and the amplitude change are traced.

**Fig. 6:**
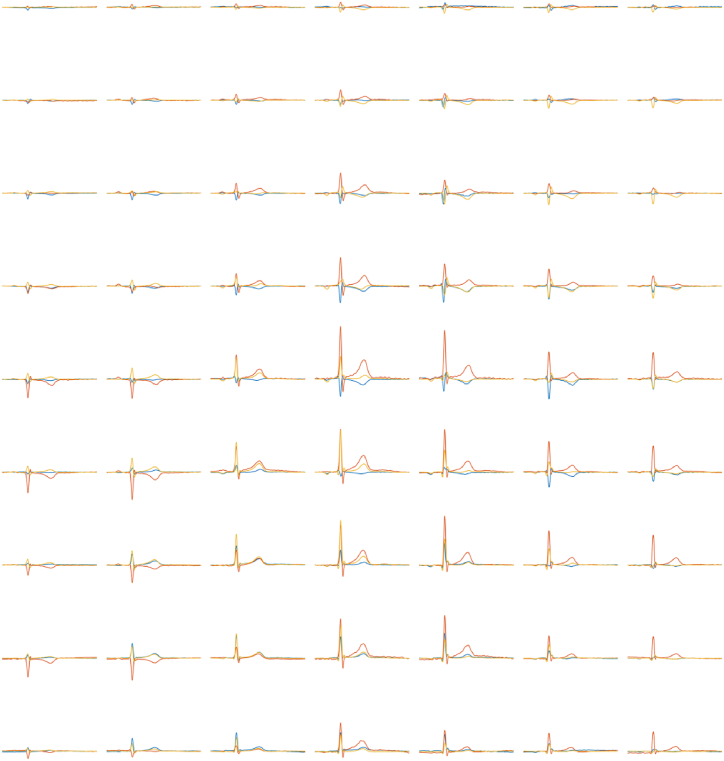
Heart signals with time for each point. The blue line represents the x direction, red represents the y direction, and yellow represents the z direction.

Due to the asynchronous measurement for each line, electrocardiogram (ECG) signals were collected synchronously with MCG signals as reference signals to achieve MCG data registration. Fig. 6 demonstrates the vector MCG signals in a cardiac cycle for each detection point. Each cycle lasted for 967 ms, as the heart rate was 62 beats per minute. The vertical coordinate range was fixed from 50 pT to 100 pT for the signals in all detection points. The blue line represents the x direction, red represents the y direction, and yellow represents the z direction in Fig. 6.

**Fig. 7:**
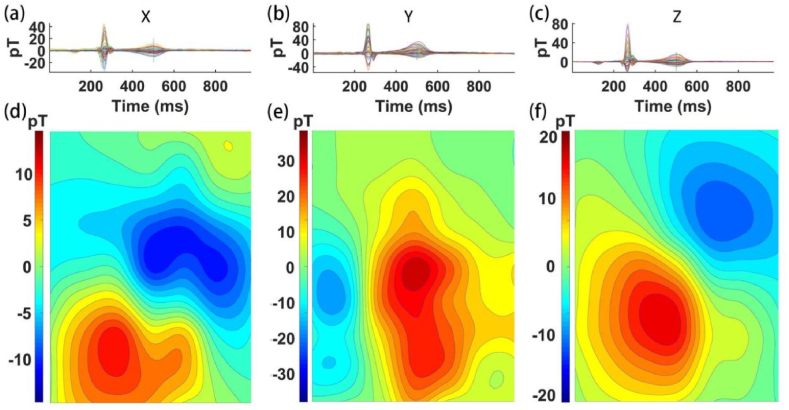
(a) ∼ (c) are respectively the overlayed time-domain MCG signals for all measuring points in the x, y, and z directions; (d) to (f) are sequentially the magnetic flux density maps at the T-peak positions.

**Fig. 8:**
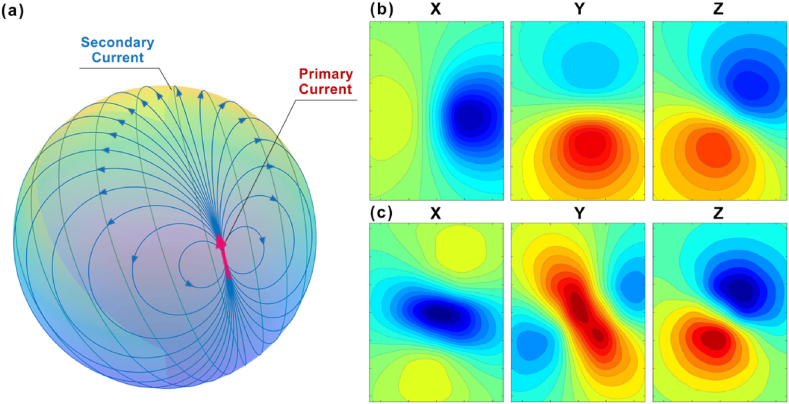
(a) Is a simple bio-electric current model; (b) and (c) simulate the magnetic flux density. maps without and with the secondary current, respectively.

Fig. 7 (a) ∼ (c) individually illustrate the time-domain overlayed graphs of MCG signals obtained from all test points along the x, y, and z axes. Capturing the magnetic field data at the T-peak locations allows for the generation of magnetic flux density maps in the x, y, and z directions, which are depicted in Fig. 7(d) ∼ (f). Generally, the $B_{z}$ map is considered an important measurement for the diagnosis of cardiac diseases [25]. Here, a typical bipolar distribution was found for $B_{z}$. The $B_{x}$ and $B_{y}$ maps show quite different density distributions, providing the possibility of finding more information for diagnosis. Herein, a simple bio-electric current model was built, as shown in Fig. 8 (a). A sphere was used to approximate the heart, where the bio-electric current was distributed on the surface. The magnetic flux density above the sphere was simulated.

In real cardiac electrophysiological processes, both primary and secondary currents exist [26]. FIG.8 (b) shows the simulation result without the secondary current, and FIG.8 (c) shows the results including the primary and secondary currents. The maps of $B_{z}$ are similar, so it seems that this information was not enough to distinguish the real current distribution. However, the maps of $B_{x}$ and $B_{y}$ show a great difference, thereby providing more information that can be used to find the real current distribution. Interestingly, the measured results shown in FIG.7 are similar to those shown in FIG.8 (c), which is closer to the real situation. It carries substantial promise in diagnosing heart diseases and offering a more profound understanding of cardiac problems' inversion. A previous report showed that vector MCG can be used to analyze the spatial structure of the current density distribution in the heart, which allows high accurate diagnosis of myocardial ischemia as well as myocardial damage in patients who have recovered from COVID-19 [27]. It was also reported that vector MCG could be used for the standardization of the fMCG waveforms and that it can provide a more complete and accurate analysis of fetal arrhythmias [16].

**Fig. 9:**
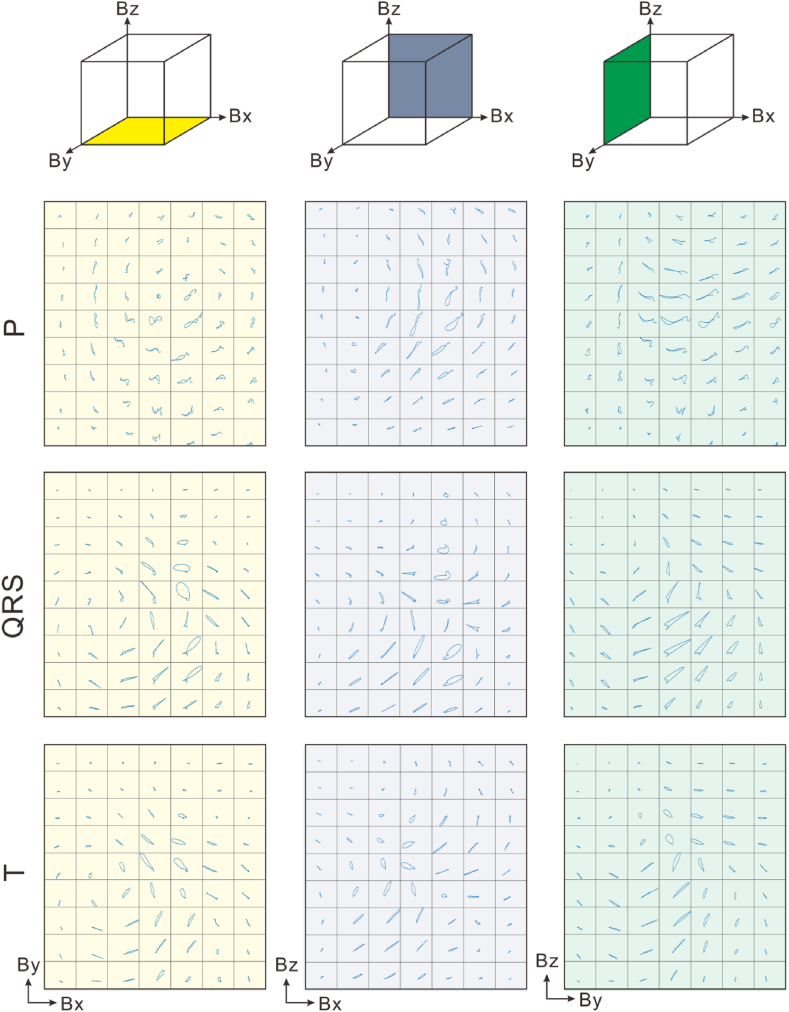
7 × 9 maps of the 3-direction projection of the heart signal scatter plot.

In a further step, the scatter plot was projected onto x-y, x-z, and y-z planes. In Fig. 9 and 7 × 9 maps with 3-direction projections are demonstrated. Since the P peak, QRS peak, and T peak differ greatly in amplitude, the scatter plot was divided into 3 parts in order to distinguish the information from the 3 stages. Interestingly, vortex patterns could be observed, thereby providing another approach to diagnosis.

## 3 Conclusion

### 3.1 Research conclusion

In conclusion, a compact-size SERF magnetometer was developed, which showed a sensitivity of around 25 fT/ $\sqrt{\text{Hz}}$. Utilizing these magnetometers, a multichannel vector MCG system was built, in which sensor manipulation and synchronous heart signal acquisition were achieved through our control software. In order to obtain the triaxial heart signal, two sensors were arranged orthogonally, and the scanning result was a 7 × 9 vector MCG map. For each detection point, it was possible to measure high-quality time-domain vector signals due to the sufficiently high sensitivity of the magnetometers. To demonstrate the vectorial information, a vector scatter plot form was also provided. In addition, a basic simulation of the magnetic field generated by bio-electric currents is carried out. The results indicated that in our model, the single z-direction MCG measurements exhibited limited sensitivity in reflecting the secondary currents, suggesting that traditional uniaxial MCG testing may not fully capture the cardiac bio-electric current situation. However, the triaxial MCG measurements, particularly the magnetic field information in the x and y directions, demonstrated greater sensitivity to the secondary currents. This finding holds significant potential for diagnosing cardiac diseases and providing deeper insights into the inversion of cardiac issues.

### 3.2 Future works and research limitations

In this work, we have achieved the measurement of triaxial MCG, which basically meets the technical requirements of commercial instruments. Although a basic bio-electric current model can demonstrate that triaxial MCG measurements can reflect more spatial current information, further empirical support is needed to validate its diagnostic effectiveness for cardiac diseases. We are currently working on a project that utilizes deep learning AI algorithms to classify heart health conditions, and the results of this work provide various presentations of triaxial MCG data, which is beneficial for the algorithm to make more accurate judgments. Meanwhile, we are also collaborating with medical institutions to conduct clinical case studies, analyzing MCG abnormalities in combination with the bio-magnetic vector model reflecting cardiac electrophysiological activities.

## Funding

This research was funded by the 10.13039/100014717National Natural Science Foundation of China [U20A20219].

## Data availability statement

The data that support the findings of this study are available on request from the corresponding author. Relevant data is also deposited into a publicly available repository, the address is https://pan.zjut.edu.cn/share/c153f92bd0f93f1d1a81935c82.

## CRediT authorship contribution statement

**Shengran Su:** Investigation. **Zhenyuan Xu:** Software. **Xiang He:** Visualization, Data curation. **Guoyi Zhang:** Visualization, Methodology. **Haijun Wu:** Software. **Yalan Gao:** Investigation. **Yueliang Ma:** Formal analysis. **Chanling Yin:** Data curation. **Yi Ruan:** Resources. **Kan Li:** Supervision. **Qiang Lin:** Supervision, Funding acquisition.

## Declaration of competing interest

The authors declare that they have no known competing financial interests or personal relationships that could have appeared to influence the work reported in this paper.
